# Correlates of Health Literacy among Farmers in Northern Thailand

**DOI:** 10.3390/ijerph17197071

**Published:** 2020-09-27

**Authors:** Hannah Montgomery, Siena Morgan, Kriengkrai Srithanaviboonchai, Pisittawoot Ayood, Penprapa Siviroj, Michele M. Wood

**Affiliations:** 1 Department of Public Health, California State University Fullerton, 800 North State College Boulevard, Fullerton, Fullerton, CA 92834, USA; hmontgomery@fullerton.edu (H.M.); sienalmorgan@gmail.com (S.M.); mwood@fullerton.edu (M.M.W.); 2 Faculty of Medicine, Chiang Mai University, 110 Intavaroros, Sriphum, Muang, Chiang Mai 50200, Thailand; psiviroj@gmail.com; 3 Research Institute for Health Sciences, Chiang Mai University, 110 Intavaroros, Sriphum, Muang, Chiang Mai 50200, Thailand; 4 Sankamphaeng Hospital, Buak Khang Sub-district, Sankamphaeng, Chiang Mai 50130, Thailand; p_ayood@yahoo.com

**Keywords:** health literacy, farmers, health education, health communication, Thailand

## Abstract

Low health literacy is a barrier to public health efforts worldwide. Agricultural workers have an elevated risk for lower health literacy, with important health implications because of their potential exposure to harmful chemicals. The Asian Health Literacy Survey (AHLS) has been developed and translated for use in several different Asian countries and is standardized for easy comparisons across regions. However, it has not been translated for use in Thailand. The purpose of this study was to (1) to determine the health literacy of rural Thai farmers in Northern Thailand, and (2) identify correlates of health literacy within this group. Internal consistency of the Thai AHLS translation was “excellent” (alpha = 0.92). Descriptive results showed that health literacy was relatively high (M = 34.98/50, SD = 6.87). Education, income, working as a village health volunteer, age, length of time farming, no chemical use in farming, health, and pesticide screening were statistically significant correlates of health literacy (*R*^2^ = 0.19). Thai farmers had higher health literacy than reported for several other Asian countries. Results may be used to inform the design of future health promotion programs.

## 1. Introduction

Having sufficient health literacy is important when individuals manage health problems, as well as in everyday life [1]. For optimal health, people “need to be able to find, understand, and use health information and services” [2]. Individuals with low health literacy may struggle navigating health care systems, especially when unfamiliar with medical terms, how bodies function, how to interpret numbers or risk to make healthcare decisions, and when they have little formal education or complex conditions that require extensive care. Ultimately, poor health literacy can have negative effects on health, resulting in poor outcomes such as disease progression, poorer overall health status, and higher rates of hospitalization and emergency services use [3,4,5]. Identifying correlates of low health literacy can help practitioners design more effective health education programs.

Many individuals have limited ability to understand and apply health information. Title V of the United States 2010 Patient Protection and Affordable Care Act (p. 518) defines health literacy as “the degree to which an individual has the capacity to obtain, communicate, process, and understand basic health information and services to make appropriate health decisions.” Health literacy involves skills related to numbers, such as understanding nutrition labels and test results, as well as basic skills regarding health knowledge, such as identifying credible sources of information [6]. Not understanding basic concepts can make it difficult to understand printed and verbal communications about one’s own health.

Low health literacy also can lead to difficulties navigating health care systems [7]. These include under-use or nonuse of healthcare services, improper management of chronic diseases and illnesses, and inappropriate use of medications. Consequently, individuals may not know how to manage health crises, which can exacerbate the effects of poor health and chronic conditions.

Language barriers can lead to incomplete understanding of physician-provided information, contributing to patient dissatisfaction and other negative outcomes. Many people have difficulty reading/understanding medication and instruction labels, including warnings [8]. In the United States, these problems may be heightened because health information in languages other than English is often limited, and translations are not always accurate. Multiple studies have documented negative health consequences of such language barriers [9]. Negative outcomes may be related to underuse of health care services, such as too few clinic and follow-up visits. Other consequences involve overuse of services, such as unnecessary or prolonged outpatient and emergency room visits.

Low health literacy has been linked to poor physical and mental health, with negative impacts on the diagnosis and treatment of disease [10]. Low health literacy can contribute to low self-esteem, financial drain, and societal inequity, all of which can lead to negative health consequences [8].

Public health and medical professionals bear responsibility for increasing patient health literacy. Unfortunately, health promotion materials are often developed without considering the reading level or health literacy of intended audiences [6]. Even individuals with strong health literacy may struggle with challenging tasks such as interpreting quantitative information, interpreting risks, and making health care decisions. These challenges may be even more daunting when people are frightened or confused, and when they have complex conditions [11].

Health literacy was originally studied primarily in the United States and Canada, and subsequently expanded to other high-income countries such as the United Kingdom, the Netherlands, Switzerland, Australia, Japan, Korea, and Taiwan [1]. Health literacy research also has been conducted in lower-middle income countries, such as Indonesia and Vietnam, and in upper-middle income countries, such as Malaysia and Kazakhstan [12].

Low health literacy is common in high-income countries, such as the United States, and is even more prevalent in low- and middle-income countries [13]. Thailand has made rapid economic advancements, and according to the World Health Organization is now considered an upper-middle income country [14]. Health literacy research in countries in transition, such as Thailand, can help inform health promotion efforts, leading to improved health outcomes and greater health equity [15].

For example, in a recent study, health literacy among women living in a migrant worker community on the Myanmar-Thailand border was assessed as “low”, with just over a third (37%) demonstrating adequate health literacy despite nearly two-thirds indicating they were literate (63%). In this study, level of education was a greater predictor of health literacy than reading literacy. Researchers identified the critical role of verbal communication and audiovisual messaging for improving health outcomes among this group [16]. Research examining elders living in Bangkok concluded that “limited” health literacy was common among Thai elders, and was significantly associated with occupational history, lower education, lower vision, and lower reading ability. These authors emphasized the importance of developing health interventions informed by an understanding of local health literacy [15].

Groups that are more likely to have poor health literacy include individuals with lower socioeconomic status, lower educational attainment, and lower reading/writing ability; general labor workers, agricultural or rural migrant occupation workers, older adults, racial/ethnic minorities, and people with compromised health status [6,15,16,17,18,19,20,21,22]. Adequate health literacy is especially important for individuals in occupations that may involve exposure to dangerous chemicals [23,24]. Research has shown a link between low heath literacy and unsafe pesticide use among farmers, highlighting the importance of efforts to increase health literacy among such workers [21,22]. Thus, farmers in Thailand, who have been identified as having incomes and education below the national average and who face potential chemical exposure, represent a particularly important potential risk group [25].

Prior research has assessed health literacy in a variety of ways. One major European study used the Test of Functional Health Literacy in Adults (TOFHLA) to measure overall health literacy among patients in outpatient settings in two public teaching hospitals [26]. Results indicated that only half the participants answered more than 80% of the questions correctly, 15% of patients could not understand basic prescription information printed on bottles, and 37% could not understand instructions about taking medication on an empty stomach [26]. Research examining health literacy among a nationally representative sample of US adults 16 years of age and older who spoke English or Spanish, including those in prison, found that 14% had “below basic”, 22% had “basic”, 53% had “intermediate”, and 12% had “proficient” health literacy [27]. More recent pilot research conducted in the United States measured health literacy among a nationally representative internet panel of adults using the Health Literacy Skills Instrument, with a different set of outcome categories [28]. In all, 38% of participants had “below basic”, 22% had “basic”, and only 40% had “proficient” health literacy.

Developing standardized health literacy scales and translating them into multiple languages can help advance health literacy research. Unfortunately, relatively fewer scales have been designed for use in Asia compared to other regions. An exception is the Health Literacy Survey (HLS-EU-Q47), which was developed for use in Europe and then translated as part of a population-based study surveying residents of six Asian countries in seven languages: Indonesian, Kazakh, Russian, Malay, Myanmar/Burmese, Mandarin, and Vietnamese [12]. Translated questionnaires were pilot-tested and evaluated by experts from each country, with standardized scoring to enable comparisons. Thus far, the AHLS has not been translated and tested for use in Thailand, however. The present study extends this work to Thailand by translating the AHLS and administering it to rural Thai residents.

The purpose of this study was to: (1) to determine the health literacy of rural Thai farmers in Northern Thailand, and (2) identify correlates of health literacy within this group.

## 2. Materials and Methods

### 2.1. Study Design

The study used a descriptive-analytic cross-sectional survey design. Multivariate analysis was used to control for confounding.

### 2.2. Setting

This project examined farmers in the On Tai subdistrict, Sankampaeng district, Chiang Mai province in northern Thailand. The area is typical of rural farming communities of northern Thailand. Data were collected via face-to-face interviews.

### 2.3. Participants

A volunteer sample was recruited. To be eligible, individuals must have been: (1) at least 18 years of age, (2) residents of the On Tai subdistrict (Tambon), (3) able to speak Thai, and (4) currently employed as farmers. Recruitment was conducted through the local community health center registry list of farmers from all 11 villages in Tambon On Tai. There were 405 registered farmers, all of whom were invited to participate. Of those invited, 344 volunteered (85%), and all completed the questionnaire.

### 2.4. Measures

The study collected health literacy information via a Thai translation of the Asian Health Literacy Survey (AHLS) and several demographic items.

#### 2.4.1. AHLS

The AHLS (HLS-EU-Q47) was originally created to measure health literacy in European countries and was later translated and validated for Asian populations [12,29]. In this study, AHLS items were translated to Thai by members of the CMU Faculty of Medicine who speak and write in Thai and English, using the translation-back translation method [30] (See Table A1).

The instrument consisted of 47 items and measured four competencies (access/obtain, understand, appraise/judge/evaluate, and apply/use health information) that fell within three domains (health care, disease prevention, and health promotion). Each item was rated on an ordinal scale ranging from 1 to 4 (1 = very difficult, 2 = difficult, 3 = easy, 4 = very easy). An overall health literacy score was calculated using the formula provided by Duong and colleagues [12]: Index = (mean − 1) × (50/3). Standardized scores ranged from 0 to 50, with 0 representing the lowest level of health literacy and 50 representing the highest. To reduce bias, only data for individuals who completed at least 42 of 47 items (~90%) were included in the analysis.

#### 2.4.2. Demographics

Participant demographics were collected, including sex, age, religion, marital status, education level, income, household size, farming practices, and overall health status [31].

### 2.5. Data Collection Procedures

Data were collected by Chiang Mai University (CMU) medical students and Research Institute for Health Science research staff, under the supervision of study investigators. Training consisted of study protocol review, item-by-item questionnaire review, and supervised roleplay. After questionnaire translation and interviewer training were conducted, the questionnaire was pre-tested among a small group of farmers from outside the study area.

Farmers were invited to participate by word of mouth and instructed to gather in a central location within the village at a designated time. Interviewers met with participants privately, described the study, obtained verbal informed consent, and then conducted the interview, which took approximately 30 min. Data collection was anonymous. A small gift valued under $5 was offered to participants.

### 2.6. Data Management and Analysis

Data were analyzed using SPSS version 26.0. Standard multiple linear regression was used to identify correlates of health literacy. For categorical variables included in the regression analysis, the reference groups were those who: had lower education, had lower income, were not village health volunteers, were female, used chemicals in farming, rated their health as other than very good (i.e., very bad, bad, moderate, or good), had not been screened for pesticides, and had less farming experience (i.e., 20 years or fewer). Information from a small number of participants who elected to skip some items was excluded from the analysis.

### 2.7. Ethical Considerations

Procedures were implemented in accordance with the Helsinki Declaration of 1975. The study was a collaboration between investigators from Chiang Mai University (CMU) in Thailand (#COM-2560-047911-4791, 7/26/17) and California State University, Fullerton (CSUF) in the United States (#HSR-17-0193, 6/21/17), and was approved by Institutional Review Boards at both institutions. Verbal informed consent was collected and documented by interviewers due to the non-sensitive nature of the information collected.

## 3. Results

### 3.1. Participant Characteristics

Participant characteristics are reported in Table 1. Age ranged from 35 to 84 years (M = 60.59, SD = 7.07). The median number of years having worked as a farmer was 30, ranging from 1 to 70 years. In general, participants had fairly low levels of education and income, and about half worked in more than one job. All but 2% attended at least some school (6/343). Half reported having another form of employment in addition to farming (175/336, 52%). Of these, nearly half (163/336, 48%) had some general type of employment, including working as a daily laborer, in construction, and with handicraft. About a third reported they owed money and did not have enough to live their lives (124/343, 36%); about a quarter (74/343, 22%) reported having more than enough to live their lives with the ability to save money.

In the prior 12 months, about two-thirds of participants reported using herbicides (229/341, 67%) and pesticides (224/341, 66%), about a quarter used fungicides (89/341, 26%), while others reported using no chemicals in their farming (67/341, 20%). Most used fertilizer (333/340, 98%); about half reported using both chemical and organic fertilizers (171/333, 51%). More than half indicated they had been screened for pesticides in the past (223/341, 65%), most of whom had been screened within the previous 5 years (189/209, 90%). Of those tested, about half reported blood screening levels that were “normal” (115/223, 52%). Reasons given for not being screened included not knowing the test was available, not having time, not using pesticides, lacking interest, not going to the doctor, being afraid of needles, and not believing they were at risk.

The majority of participants had Universal Coverage Insurance (261/339, 77%), and about half reported having a chronic health condition (156/335, 47%). Chronic conditions included hypertension (107/338, 32%), high cholesterol (43/338, 13%), diabetes (35/339, 10%), cardiovascular disease (4/338, 1%), renal failure (6/339, 2%), and others (49/338, 14%). When asked to rate their health, the vast majority (322/240, 95%) rated it as “very good” to “moderate.” Most respondents reported prior participation in village meetings (329/341, 96%), and some were village health volunteers (38/340, 11%). (In Thailand, village health volunteers are members of the community who volunteer and are trained to distribute basic health information to community residents.)

### 3.2. Asian Health Literacy Survey

The internal consistency reliability for the Thai AHLS was excellent (Cronbach’s alpha = 0.92). The overall mean AHLS score for the sample was 3.10 (SD = 0.41), and the standardized AHLS score [12] was 34.98 (SD = 6.87). Data were fairly complete. Most items (42/47) were skipped by 3 or fewer respondents (<1%). One exception was the question, “On a scale from very easy to very difficult, how easy would you say it is to judge how information from your doctor applies to you?”, which was skipped by 9 of 344 total respondents (<3%). The next most frequently skipped item was, “… how easy would you say it is to understand the leaflets that come with your medicine?”, which was skipped by 5 respondents (<2%). Although even the lowest response rates are still fairly high, they may reflect items that were more difficult for respondents to understand and answer.

Table 2 presents responses for each AHLS competency subarea. For assessing and obtaining health information, finding information about reducing noise and pollution (69%) was the competency most frequently rated as “fairly easy” or “very easy.” For understanding health information, the competencies of understanding doctor and pharmacist instructions on how to take prescribed medicine (96%) and understanding what doctors say (96%) were most frequently rated as fairly/very easy. For appraising, judging, evaluating health information, the competency most frequently rated as fairly/very easy was judging how housing conditions help one stay healthy (93%). For the ability to apply and use health information, following medication instructions (97%) was most frequently rated as fairly/very easy (95%).

### 3.3. Correlates of Health Literacy

A standard multiple linear regression was conducted to determine if education, income, having worked as a village health volunteer, sex, age, length of time working as a farmer, use of chemicals in farming, health rating, and pesticide screening were significant correlates of overall health literacy. Results indicated a statistically significant proportion of variation in health literacy (19%) was predicted by the variables in the model, *F*(9, 322) = 8.23, *p* < 0.001, suggesting a medium to large effect. Specifically, those with higher education, higher income, experience working as a health volunteer, younger age, more experience farming, no use of chemicals in farming, better self-rated health, and prior screening for pesticide exposure had higher general health literacy, controlling for the other variables in the model. Sex was not statistically significantly correlated with health literacy (See Table 3).

## 4. Discussion

Cronbach’s alpha (0.92) indicated the translated AHLS had excellent internal consistency. The overall standardized AHLS mean score of Thai farmers in this study was 34.98. Compared to prior research, study participants had a higher standardized health literacy score than those in Indonesia (31.4), Kazakhstan (31.6), Malaysia (32.9), Myanmar (31.3), Taiwan (34.4), and Vietnam (29.6) [12]. Significant correlates of higher health literacy included more education, higher income, experience working as a village health volunteer, younger age, more experience farming, no chemical use in farming, better perceived health, and prior pesticide screening. These findings are consistent with prior research identifying lower education and older age as important risk factors contributing to lower health literacy, and the use of chemicals in farming and compromised health status as outcomes [15,16,17,18,19,20,21,22]. The fact that lower health literacy was associated with not having been screened for pesticide exposure, that 20% of respondents reported blood test screening results considered “at risk” or “unsafe/dangerous”, and that another 18% were unsure of their results reinforces the need for developing and implementing interventions designed to increase health literacy among farmers [23,24]. Results contradict prior research that found lower age associated with lower health literacy [22].

The majority of health literacy research has been conducted with English-speaking populations. Relatively fewer studies have focused solely on measuring general health literacy, and not all instruments are appropriate for all settings. For example, the TOFHLA focuses on reading comprehension for healthcare and medical terms, but it is somewhat more complex and structured as a self-administered test, making it more challenging to use with rural populations [26]. The AHLS is relatively easy to use and has been translated into several Asian languages, but previously had not been translated to Thai. The present study helps address this gap.

There are several implications of this research. The results can be used by local community leaders and health care professionals to tailor health literacy education efforts, which can lead to improved health promotion programs and overall quality of care. Specifically, health literacy efforts should target those who: have lower education, have lower income, are older, use chemicals in their farming, have not been tested for pesticide exposure, have less experience farming, rate their health as poorer, and have not worked as a village health volunteer. Although participants had an overall higher standardized health literacy score compared to scores reported for other Asian countries, their health literacy can continue to improve. This is especially true for those identified as potential targets of health literacy promotion efforts, having characteristics correlated with lower health literacy. Improved health literacy can help people overcome barriers they face when navigating the healthcare system, resulting in more effective health promotion programs and better community health [12].

The current study adds knowledge to a growing research area in Thailand. Health care professionals may use results to improve current programs and overall farmer health. The study had a high response rate; 344 of 405 registered farmers participated (85%); thus, the sample can be viewed as an approximate representation of the farmer population in this subdistrict, with potential self-selection bias. Consistent with prior research in six other Asian countries, the Thai translation of the AHLS showed high internal consistency [12]. It should be noted that study participants represent farmers who were recruited from a specific region in Northern Thailand, and results cannot be generalized to the entire country. Although the sample reported lower levels of formal education, clinic staff reported that this group of farmers had participated in prior health-related research studies, and their health literacy may have benefitted from these experiences.

Future health literacy research should include other regions and other populations in Thailand to provide a more complete picture nationwide. The AHLS should be translated to other languages, and additional research should be conducted to better understand health literacy differences across Asia, as well as globally, so these differences may be addressed more effectively.

## 5. Conclusions

This study advances prior health literacy research by translating the AHLS to Thai and testing it among residents of rural Thailand. The translation demonstrated excellent internal consistency and should be tested in other Thai communities. Future health literacy efforts in this community should target those who have lower education, have lower income, are older, use chemicals in their farming, have not been tested for pesticide exposure, have less experience farming, rate their health as poorer, and have not worked as a village health volunteer.

## Figures and Tables

**Table 1:** Participant Characteristics (*N* = 344) ^1^.

Characteristic	*n*	%
Sex		
Male	221	65
Female	120	35
Age		
35–44 years	10	3
45–54 years	52	15
54–64 years	205	60
65–74 years	65	19
75 + years	12	3
Religion		
Buddhist	335	97
Christian	8	2
Other	1	1
Marital status		
Single	14	4
Married	299	87
Divorced	10	3
Widowed	19	6
Education Level		
No school; cannot read/write	4	1
No school; can read/write	2	1
Primary school	279	81
Secondary school	24	7
High school/vocational school	30	9
University or higher	4	1
Annual Income (Baht)		
฿0–50,000	234	68
฿50,001–100,000	74	21
฿100,001–200,000	27	8
฿200,000+	9	3
Household Size		
1	13	4
2	82	24
3	87	25
4	55	16
5	59	17
6+	47	14
Fertilizer use		
Both chemical and organic	171	51
Chemical only	127	38
Organic only	28	9
No fertilizer use	7	2
Screened for pesticides		
Yes	223	65
No	118	35
Pesticide screening results		
Normal	115	52
Safe	23	10
At risk	43	19
Unsafe/dangerous	2	1
Don’t know/unsure	40	18
Self-rated health		
Very good	50	15
Good	107	32
Moderate	165	48
Bad	18	5
Very bad	0	0

^1^ Some participants skipped one or more items.

**Table 2:** Thai Asian Health Literacy Survey (AHLS) Response Frequencies (*N* = 344) ^1^.

Survey Item	% (*n*)								
	*N*	Very Difficult		Fairly Difficult		Fairly Easy		Very Easy	
**Assessing/Obtaining Health Information (Subarea 1)**									
Find information (such as reducing noise and pollution, creating green spaces, leisure facilities) on how your neighborhood could be more health-friendly?	342	9	(31)	22	(76)	35	(118)	34	(117)
Find information on healthy activities such as exercise, healthy food and nutrition?	342	13	(45)	21	(71)	32	(109)	34	(117)
Find out where to get professional help (such as doctor, pharmacist, psychologist) when you are ill?	342	18	(63)	16	(54)	29	(100)	37	(125)
Find out about activities (such as meditation, exercise, walking, Pilates etc.) that are good for your mental well-being?	342	12	(40)	24	(83)	30	(103)	34	(116)
Find information about how to manage unhealthy behavior such as smoking, low physical activity and drinking too much?	342	13	(44)	24	(81)	33	(114)	30	(103)
Find out about efforts to promote your health at work?	341	14	(48)	25	(85)	36	(122)	25	(86)
Find information on how to manage mental health problems like stress or depression?	342	16	(54)	26	(89)	34	(117)	24	(82)
Find information on how to prevent or manage conditions like being overweight, high blood pressure or high cholesterol?	342	15	(51)	27	(93)	32	(110)	26	(88)
Find information about vaccinations and health screenings (such as breast exam, blood sugar test, blood pressure) that you should have?	342	24	(81)	28	(97)	26	(90)	22	(74)
Find out about political changes (such as legislation, new health screening programs, change of government, restructuring of health services etc.) that may affect health?	342	29	(100)	24	(82)	25	(85)	22	(75)
Find out what to do in case of a medical emergency?	341	29	(98)	28	(95)	24	(82)	19	(66)
Find information about symptoms of illnesses that concern you?	341	33	(112)	28	(95)	24	(83)	15	(51)
Find information on treatments of illnesses that concern you?	341	30	(101)	33	(113)	24	(81)	13	(46)
**Understanding Health Information (Subarea 2)**									
Understand your doctor’s or pharmacist’s instruction on how to take a prescribed medicine?	342	0	(0)	4	(12)	19	(65)	77	(265)
Understand what your doctor says to you?	342	1	(2)	3	(11)	19	(65)	77	(264)
Understand health warnings about behavior such as smoking, low physical activity and drinking too much?	342	2	(6)	6	(21)	31	(105)	61	(210)
Understand information on food packaging?	342	4	(12)	5	(17)	19	(66)	72	(247)
Understand the leaflets that come with your medicine?	339	3	(11)	8	(25)	20	(69)	69	(234)
Understand advice on health from family members or friends?	342	2	(8)	9	(30)	39	(132)	50	(172)
Understand information on how to keep your mind healthy?	340	2	(9)	10	(33)	36	(121)	52	(177)
Understand why you need health screenings (such as breast exam, blood sugar test, blood pressure)?	342	2	(8)	11	(37)	38	(129)	49	(168)
Understand why you need vaccinations?	342	6	(19)	10	(36)	33	(112)	51	(175)
Understand information in the media (such as Internet, newspaper, magazines) on how to get healthier?	341	7	(22)	16	(55)	33	(114)	44	(150)
Understand what to do in a medical emergency?	341	9	(29)	19	(66)	35	(119)	37	(127)
**Appraising/Judging/Evaluating Health Information (Subarea 3)**									
Judge how your housing conditions help you to stay healthy?	341	1	(5)	6	(20)	25	(85)	68	(231)
Judge how reliable health warnings are, such as smoking, low physical activity and drinking too much?	342	<1	(2)	7	(24)	43	(146)	50	(170)
Judge which everyday behavior (such as drinking and eating habits, exercise etc.) is related to your health?	341	2	(8)	7	(24)	30	(103)	61	(206)
Judge when you need to go to a doctor for a check-up?	342	2	(8)	9	(31)	32	(108)	57	(195)
Judge how where you live (such as your community, neighborhood) affects your health and well-being?	341	2	(8)	10	(33)	26	(89)	62	(211)
Judge how information from your doctor applies to you?	335	6	(20)	16	(52)	42	(141)	36	(122)
Judge the advantages and disadvantages of different treatment options?	342	8	(28)	20	(68)	40	(135)	32	(111)
Judge if the information on health risks in the media (such as TV, Internet or other media) is reliable?	342	9	(31)	24	(81)	36	(122)	31	(108)
Judge which health screenings (such as breast exam, blood sugar test, blood pressure) you should have?	341	12	(39)	24	(81)	31	(107)	33	(114)
Judge if the information about illness in the media (such as TV, Internet, or other media) is reliable?	342	12	(40)	29	(98)	39	(134)	20	(70)
Judge when you may need to get a second opinion from another doctor?	341	22	(74)	19	(66)	34	(115)	25	(86)
Judge which vaccinations you may need?	342	25	(84)	27	(91)	24	(83)	24	(84)
**Applying/Using Health Information (Subarea 4)**									
Follow the instructions on medication?	342	<1	(1)	3	(9)	9	(31)	88	(301)
Follow instructions from your doctor or pharmacist?	342	<1	(1)	4	(14)	16	(56)	79	(271)
Take part in activities that improve health and well-being in your community?	341	3	(10)	5	(19)	24	(81)	68	(231)
Make decisions to improve your health?	341	2	(8)	11	(37)	24	(83)	63	(213)
Use information the doctor gives you to make decisions about your illness?	341	3	(9)	12	(42)	39	(134)	46	(156)
Influence your living conditions that affect your health and well-being?	341	4	(14)	11	(38)	35	(120)	50	(169)
Decide how you can protect yourself from illness based on advice from family and friends?	340	6	(19)	11	(38)	38	(131)	45	(152)
Decide if you should have a flu vaccination?	341	8	(27)	9	(31)	29	(98)	54	(185)
Join a sports club or exercise class if you want to?	340	8	(25)	15	(52)	25	(86)	52	(177)
Call an ambulance in an emergency?	341	15	(51)	13	(44)	15	(52)	57	(194)
Decide how you can protect yourself from illness based on information in the media (such as Newspaper, leaflets, Internet or other media)?	342	9	(33)	28	(95)	38	(129)	25	(85)

^1^ Some participants skipped one or more items.

**Table 3:** Multiple Linear Regression Correlating Predictors with Health Literacy (*N* = 332).

	Model					
Variable	*B*	*SE(B)*	*β*	*t*	*95% CI*	*p*
Education (high)	2.20	0.99	0.12	2.23	0.26–4.13	0.027
Income (high)	2.64	0.80	0.18	3.28	1.03–4.22	0.001
Village health volunteer (yes)	3.80	1.16	0.17	3.28	1.52–6.08	0.001
Sex (male)	0.42	0.78	0.03	0.54	−1.11–1.94	0.593
Age (younger)	−0.12	0.06	−0.12	−2.09	−0.23–−0.01	0.038
Chemical use (no)	1.89	0.91	0.11	2.08	0.10–3.68	0.038
Health rating (very good)	2.19	1.00	0.11	2.19	0.23–4.15	0.029
Pesticide screening (yes)	2.08	0.74	0.14	2.81	0.63–3.54	0.005
Years farming (more than 20)	2.75	0.79	0.19	3.50	1.21–4.30	0.001
*R*^2^ = 0.19						

The model accounted for 19% of the variance in General Health Literacy Index Scores, *F(9, 322)* = 8.23 *p* < 0.001.

## Appendix A

**Table A1:** Health literacy questionnaire (HLS-EU-Q47).

On a Scale from Very Easy to Very Difficult, How Easy Would You Say It Is to:		Very Difficult	Fairly Difficult	Fairly Easy	Very Easy
Q 1	find information about symptoms of illnesses that concern you?	1	2	3	4
Q 2	find information on treatments of illnesses that concern you?	1	2	3	4
Q 3	find out what to do in case of a medical emergency?	1	2	3	4
Q 4	find out where to get professional help (such as doctor, pharmacist, psychologist) when you are ill?	1	2	3	4
Q 5	understand what your doctor says to you?	1	2	3	4
Q 6	understand the leaflets that come with your medicine?	1	2	3	4
Q 7	understand what to do in a medical emergency?	1	2	3	4
Q 8	understand your doctor’s or pharmacist’s instruction on how to take a prescribed medicine?	1	2	3	4
Q 9	judge how information from your doctor applies to you?	1	2	3	4
Q 10	judge the advantages and disadvantages of different treatment options?	1	2	3	4
Q 11	judge when you may need to get a second opinion from another doctor?	1	2	3	4
Q 12	judge if the information about illness in the media (such as TV, Internet, or other media) is reliable?	1	2	3	4
Q 13	use information the doctor gives you to make decisions about your illness?	1	2	3	4
Q 14	follow the instructions on medication?	1	2	3	4
Q 15	call an ambulance in an emergency?	1	2	3	4
Q 16	follow instructions from your doctor or pharmacist?	1	2	3	4
Q 17	find information about how to manage unhealthy behavior such as smoking, low physical activity and drinking too much?	1	2	3	4
Q 18	find information on how to manage mental health problems like stress or depression?	1	2	3	4
Q 19	find information about vaccinations and health screenings (such as breast exam, blood sugar test, blood pressure) that you should have?	1	2	3	4
Q 20	find information on how to prevent or manage conditions like being overweight, high blood pressure or high cholesterol?	1	2	3	4
Q 21	understand health warnings about behavior such as smoking, low physical activity and drinking too much?	1	2	3	4
Q 22	understand why you need vaccinations?	1	2	3	4
Q 23	understand why you need health screenings (such as breast exam, blood sugar test, blood pressure)?	1	2	3	4
Q 24	judge how reliable health warnings are, such as smoking, low physical activity and drinking too much?	1	2	3	4
Q 25	judge when you need to go to a doctor for a check-up?	1	2	3	4
Q 26	judge which vaccinations you may need?	1	2	3	4
Q 27	judge which health screenings (such as breast exam, blood sugar test, blood pressure) you should have?	1	2	3	4
Q 28	judge if the information on health risks in the media (such as TV, Internet or other media) is reliable?	1	2	3	4
Q 29	decide if you should have a flu vaccination?	1	2	3	4
Q 30	decide how you can protect yourself from illness based on advice from family and friends?	1	2	3	4
Q 31	decide how you can protect yourself from illness based on information in the media (such as Newspaper, leaflets, Internet or other media)?	1	2	3	4
Q 32	find information on healthy activities such as exercise, healthy food and nutrition?	1	2	3	4
Q 33	find out about activities (such as meditation, exercise, walking, Pilates etc.) that are good for your mental well-being?	1	2	3	4
Q 34	find information (such as reducing noise and pollution, creating green spaces, leisure facilities) on how your neighborhood could be more health-friendly?	1	2	3	4
Q 35	find out about political changes (such as legislation, new health screening programs, change of government, restructuring of health services etc.) that may affect health?	1	2	3	4
Q 36	find out about efforts to promote your health at work?	1	2	3	4
Q 37	understand advice on health from family members or friends?	1	2	3	4
Q 38	understand information on food packaging?	1	2	3	4
Q 39	understand information in the media (such as Internet, newspaper, magazines) on how to get healthier?	1	2	3	4
Q 40	understand information on how to keep your mind healthy?	1	2	3	4
Q 41	judge how where you live (such as your community, neighborhood) affects your health and well-being?	1	2	3	4
Q 42	judge how your housing conditions help you to stay healthy?	1	2	3	4
Q 43	judge which everyday behavior (such as drinking and eating habits, exercise etc.) is related to your health?	1	2	3	4
Q 44	make decisions to improve your health?	1	2	3	4
Q 45	join a sports club or exercise class if you want to?	1	2	3	4
Q 46	influence your living conditions that affect your health and well being?	1	2	3	4
Q 47	take part in activities that improve health and well-being in your community?	1	2	3	4

Note: The scale was published by Duong and colleagues, Appendix A [12].
